# A Systematic Approach to Comparing Thermal Activity of the Thoracic Region and Saddle Pressure Distribution beneath the Saddle in a Group of Non-Lame Sports Horses

**DOI:** 10.3390/ani11041105

**Published:** 2021-04-13

**Authors:** Russell MacKechnie-Guire, Mark Fisher, Helen Mathie, Kat Kuczynska, Vanessa Fairfax, Diana Fisher, Thilo Pfau

**Affiliations:** 1 Centaur Biomechanics, 25 Oaktree Close, Moreton Morrell, Warwickshire CV35 9BB, UK; 2 Department of Clinical Science and Services, The Royal Veterinary College, Hawkshead Lane, Brookman’s Park, Hatfield AL9 7TA, UK; TPFau@rvc.ac.uk; 3 Woolcroft Saddlery, Mays Lane, Wisbech PE13 5BU, UK; woolcroft2002@yahoo.co.uk (M.F.); dianafisher007@yahoo.co.uk (D.F.); 4 Helen Mathie Physiotheraphy, Estate House, Matfen NE20 0RP, UK; helenmathiephysio@gmail.com; 5 Vet-IR, 83 Ducie Street, Manchester M1 2JQ, UK; kat@vet-ir.com; 6 Fairfax Saddles, The Saddlery, Fryers Road, Bloxwich, Walsall, West Midlands WS3 2XJ, UK; vanessa.fairfax@fairfaxsaddles.com

**Keywords:** magnitude, hyperthermic, hypothermic, degree, symmetry, asymmetry

## Abstract

**Simple Summary:**

Thermography is a non-invasive method for measuring surface temperatures. Due to its ease of use, it may be a convenient way of identifying hypo/hyperthermic areas under a saddle that may be related to saddle pressures. A thermal camera quantified temperatures at specific locations (left/right) of the thoracic region at three-time points; a Pliance (Novel) pressure mat determined the mean/peak saddle pressures (kPa) during a period of exercise. Differences between saddle widths in the cranial/caudal mean and peak saddle pressures were found. The maximum thermal temperatures increased post lunge and post ridden compared to the baseline. No difference between post lunge and post ridden exercise were found. The thermal activity does not appear to be representative of increased saddle pressure values. The sole use of thermal imaging for saddle fitting should be applied with caution.

**Abstract:**

Thermography is a non-invasive method for measuring surface temperatures and may be a convenient way of identifying hypo/hyperthermic areas under a saddle that may be related to saddle pressures. A thermal camera quantified minimum/maximum/mean temperatures at specific locations (left/right) of the thoracic region at three-time points: (1) baseline; (2) post lunging; (3) post ridden exercise in eight non-lame sports horses ridden by the same rider. A Pliance (Novel) pressure mat determined the mean/peak saddle pressures (kPa) in the cranial and caudal regions. General linear mixed models with the horse as the random factor investigated the time point (fixed factor: baseline; lunge; ridden) and saddle fit (fixed factor: correct; wide; narrow) on thermal parameters with Bonferroni post hoc comparison. The saddle pressure data (grouped: saddle width) were assessed with an ANOVA and Tukey post hoc comparison (*p* ≤ 0.05). Differences between the saddle widths in the cranial/caudal mean (*p* = 0.05) and peak saddle pressures (*p* = 0.01) were found. The maximum temperatures increased post lunge (*p* ≤ 0.0001) and post ridden (*p* ≤ 0.0001) compared to the baseline. No difference between post lunge and post ridden exercise (all *p* ≥ 0.51) was found. The thermal activity does not appear to be representative of increased saddle pressure values. The sole use of thermal imaging for saddle fitting should be applied with caution.

## 1. Introduction

The saddle is an essential piece of equipment coupling the horse and rider. The effects of saddle fit [1,2,3] and saddle design [1,2,3,4,5,6,7,8,9,10,11] on equine health and performance are becoming better understood. Incorrect saddle fit is thought to be a potential contributing factor in the context of back problems, poor attitude to work and poor performance [3,12,13,14,15,16,17]. One way of quantifying saddle fit is the use of an electronic pressure mat positioned beneath a saddle. This has been shown to be a reliable and accurate way to quantify saddle pressure distribution [18,19] in the static and dynamic horse [1,2,3,9,10,15,20,21,22]. Although the accuracy, repeatability and application of pressure mapping systems has been demonstrated [18,19], the costs and time associated with data collection/processing is a limiting factor for its use outside of a laboratory setting.

An association between lameness and spinal dysfunction has been reported [14,23,24,25,26,27]; however, the causal relationship between the two needs further investigations. Saddle fit should be considered as a contributing factor in this context. In saddles which had been fitted both statically and dynamically following industry guidelines, pressures in the region of the 10th–13th thoracic vertebrae were of a magnitude higher than those thought to cause back discomfort [15]. When the magnitude of pressure was reduced, with saddlery modifications, limb kinematics at trot [6], gallop [8] as well as jumping technique [7] were altered. This emphasizes the importance of saddle fit and the relationship between (high) saddle pressures and locomotor features.

Saddles are generally fitted to the horse and rider by a qualified saddle fitter who will assess the saddle both statically and dynamically. This assessment is subjective and relies on the skills and experience of the saddle fitter. This process, like other subjective assessments, e.g., visual lameness assessments [28,29,30,31,32], will likely have limitations. The subjective nature of the saddle fitting, along with the effect that saddle fit can have on the locomotor apparatus of the horse [1,2,3,5,6,7], has led to an increased availability of a variety of objective measuring systems aiming to assist with saddle fitting, potentially providing a more quantitative approach.

Thermography has been used in veterinary medicine [33], and specifically as part of a diagnostic technique when evaluating back-related conditions [34]. It has been proposed as a potential system to quantify saddle fit by evaluating thermal pattern distribution [35,36,37], providing a non-invasive diagnostic imaging technique which detects the superficial heat emission from the body by infrared radiation, indicative of the temperature of the body surface. It has been proposed that a thermal imaging camera can detect hyperthermic activity (due to friction/pressure) with more than 10 times higher sensitivity than the human hand [38].

Thermography has been used to identify focal hypothermic and hyperthermic areas in the horse and saddle [7,35,36,37,38]. Thermal symmetry of the underside of the saddle [35,36,37] and thoracic region of the horse [35,39] has been proposed to be the most important criterion [38] for determining saddle fit. The identification of focal hyperthermic areas along the midline of the thoracic spine and/or hypo/hyperthermic areas, laterally to the midline along the epaxial musculature, is thought to be indicative of incorrect saddle fit [38]. Focal hyperthermic areas are suggested to represent high friction or pressure points and hypothermic areas are suggested to represent either intense muscle spasm or severe pressure damage/swelling caused by the saddle [38]. The mean temperature differences of >2 °C between the various regions of the ventral aspect of the saddle (saddle panels) have been reported to be indicative of incorrect saddle fit and associated with saddles which showed signs of rocking/bridging [37]. Rocking/bridging could be an indication that the saddle is too narrow [2].

Thermographic assessments have been used within saddle fitting. The overarching biological assumption appears to be that areas with an increased magnitude in pressure (related to incorrect saddle fit) will alter the heat emitted from the affected region of the thoracic spine as a function of pressure. There is, however, an apparent lack of evidence specifically quantifying the association between saddle pressures (using an electronic pressure mapping system) and thermographic assessments. Initially, to determine saddle fit, a study has suggested lunging the horse for 20 min in walk, trot and canter whilst wearing a saddle (with a saddle cloth), followed by a thermal scan of the underside of the saddle and then the back [38], followed by a ridden session. It seems reasonable to expect that the thermal activity will alter as a function of exercise [40,41] hence, making it difficult to differentiate if changes in thermal activity are as a result of saddle fit or as a function of exercise [41]. To the authors’ knowledge, there are no studies which have directly quantified thermal activity and pressure distribution in relation to saddle fit in a group of horses.

The aim of this study was to quantify thermal patterns and saddle pressure distribution in a group of non-lame sports horses ridden by the same rider following a standardised exercise test. It is hypothesised that: (1) there will be differences in thermographic patterns between the baseline and a post lunge exercise test; (2) hyper/hypothermographic areas will correspond to areas where there is an increased/decreased magnitude in pressure; (3) there will be differences in thermographic patterns recorded after the lunge (without saddle) test and after the ridden exercise test; (4) there will be thermographic differences between the left and right sides of the underside of the saddle.

## 2. Materials and Methods

This study was approved by the ethics and welfare committee of the first author’s institution, project number URN 2020 1975-2. Informed, written consent was obtained prior to participation in the study. At the time of the study, the rider was free from any injuries and could withdraw their participation from the study at any point.

### 2.1. Horses

A convenience sample of eight adult elite jumping sports horses were recruited who were based at a professional show jumping facility. The inclusion criteria were that horses were free from lameness as perceived by their owners and in competitive work. Horses were of a similar type and conformation. The day preceding the study, all horses underwent a subjective veterinary assessment performed by an experienced veterinary surgeon which included visual observations in walk and trot from both the rear and lateral view as well as a palpatory examination of the thoracolumbar region. No overt signs of lameness or back discomfort/conditions were observed. On the day of data collection, the horses underwent a physiotherapy assessment assessing the presence or absence of epaxial hypertonicity and pain. In addition, each horse’s gait was quantified whilst trotting in a straight line using a validated sensor-based system (Xsens, Enschede, The Netherlands) [42].

### 2.2. Rider

One male experienced (international showjumping) rider took part in the study, height 1.72 m and a body mass 69 kg. The rider was familiar with all the horses and had been riding them regularly in the time period preceding data collection. At the time of the study, the rider was free from injury and fit to perform.

### 2.3. Saddles

On the day, saddles were assessed independently by five Society of Master Saddlers Qualified Saddle Fitters (SMSQSF). All saddles were checked for fit both statically [43] and dynamically following the Society of Master Saddlers (SMS) published guidelines [44].

### 2.4. Measuring Systems

#### 2.4.1. Thermal Imaging

A FLIR T660 hand-held camera with a standard 25° lens was used for this study. This device has a high thermal detector resolution of 640 × 480 pixels, and high thermal sensitivity or NETD (noise equivalent temperature difference) of <20 mK with a measurement accuracy of ±1–2°. The FLIR T660 is controlled for thermal drift by performing an automatic internal non-uniformity correction (NUC) when the ambient temperature changes considerably. On the day (December), a wet globe monitor was used to determine ambient temperature which ranged between 4–7 °C and humidity which ranged between 57–70%. Three thermographic scans of the thoracolumbar region were taken at 90° to the subject and at a distance of 1.5 m [45]. Scans were taken from an elevated position (caudal) to the horse. All thermograms were taken by the same experienced thermography technician. Protocols (i.e., patient preparation for scanning, enclosed area free from sunlight and draughts, scanning technique—distance and angle) were adhered to, to ensure optimal scanning conditions and thus limit artefacts on the data arising from external factors.

#### 2.4.2. Saddle Kinetics

Prior to the study, the pressure mat had been calibrated following manufacturer’s guidelines (Pliance, Novel). On the day, prior to the dynamic measurements, the pressure mat was zeroed without the saddle, girth or rider [1] and was fitted so that the pressure mat was overlaying the horse’s skin and beneath the saddle cloth and saddle as previously described [15,16,17].

### 2.5. Study Protocol

#### 2.5.1. Baseline Thermographic Scan

The horse’s rug (blanket) was removed one hour prior to baseline measurement. In this time, the horse was tied up in the stable and was not touched or groomed. Then, each horse was walked to the thermography area where baseline scans were taken.

#### 2.5.2. Standardised Unridden Dynamic Exercise (Lunge Test)

**Figure 1:**
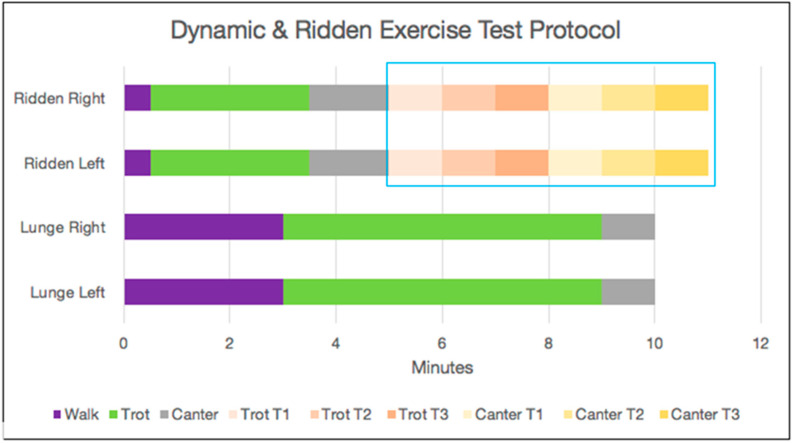
Illustrating the time spent on each rein and gait for both the dynamic unridden exercise test and ridden exercise test. The blue box represents the time when saddle pressure data were collected for trial 1 (T1), T2 and T3 on both the left and right rein in trot and canter.

Each horse underwent a standardised 20-min exercise test which consisted of the horse being lunged on a 20 m diameter circle without a saddle, in both a clockwise and anticlockwise direction for set time periods in walk, trot and canter (Figure 1). Horses were lunged by the same handler and were accustomed to the lunging environment in an indoor arena with a uniformed surface. Flat spherical cones were used to mark out the circle circumference and a central cone was positioned in the middle, representing the centre of the circle.

Figure 1—Timelines of the dynamic and ridden exercise test protocol.

After the unridden lunge test, horses were immediately walked to the scanning area. All horses’ backs were scanned following the same protocol as outlined previously. Then, horses were fitted with the Pliance saddle mat as outlined previously and prepared for riding, which included being fitted with a cotton saddle cloth, the horse’s saddle and an anatomically designed girth. No additional layers were placed beneath the saddle. Finally, each horse was walked (in-hand) to the indoor arena to perform the standardised ridden exercise test.

#### 2.5.3. Standardised Ridden Exercise Test

Each horse underwent a standardised 10-min warm-up protocol which was called by the same research technician. The warm-up protocol consisted of walk, trot and canter on both the left and right rein (Figure 1). This was followed by a prescribed ridden protocol in trot and canter, during which saddle kinetics were assessed. Data were collected during straight-line locomotion. The speed was determined using a stopwatch, with start and end points being defined by two cones positioned on the start/end of the testing track. After completion of the ridden exercise test and data collection, the horse was walked for one minute. The girth tension remained unchanged until the horse was presented for scanning. After the saddle had been removed, horses were scanned as previously described.

#### 2.5.4. Thermal Imaging of the Underside of the Saddle

Immediately after the ridden exercise test, the saddle was removed and then placed on a flat surface with the pommel and cantle resting against a vertical breeze blocked wall and a thermal scan was taken.

**Figure 2:**
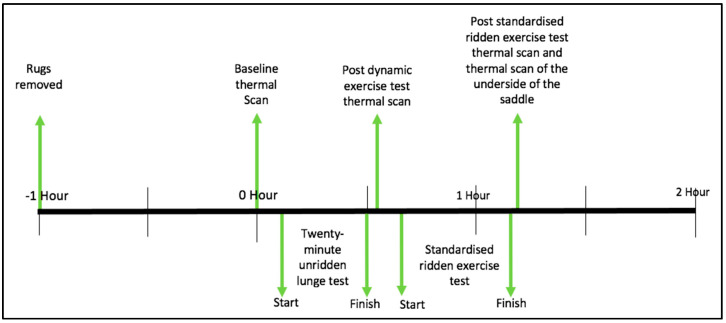
Illustrating the various stages of the experiment including the timepoints at which the thermal scans were taken along with the two exercise tests.

Figure 2—timeline illustrating the stages of the experiment.

### 2.6. Data Collection and Processing

Quantitative gait assessment—movement symmetry variables:

- minimum difference head (HDMin) and pelvis (PDMin): difference between the two minima in vertical (z) displacement observed during the two diagonal stance phases in trot;
- maximum difference head (HDMax) and pelvis (PDMax): difference between the two maxima in vertical (z) displacement observed after the two diagonal stance phases in trot;
- hip hike difference (HHD): difference between vertical upward movement amplitude of left and right tuber coxae during contra-lateral stance.

**Figure 3:**
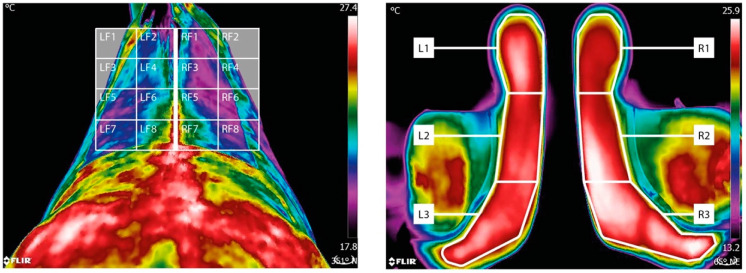
Illustrating the thermal grid reference used when quantifying thermal temperatures of the thoracic region (**left**) and ventral aspect of the saddle (**right**). A mask (grey area) was applied to areas of the grid which did not correspond to the horse’s back.

Thermographic data:

Horse: A grid reference was applied to each thermogram and minimum, maximum and mean temperatures were obtained from each location and processed using Flir software. A mask was applied to areas of the grid which did not correspond to the horse’s back (Figure 3).

Figure 3—Thermal Grid Reference for the Thoracic Region and Underneath of the Saddle.

Pooled minimum, maximum and mean temperatures (Figure 3):

- for the left cranial region defined as LF1, LF2, LF3 and LF4;
- for the right cranial region defined as RF1, RF2, RF3 and RF4;
- for the left caudal region defined as LF5, LF6, LF7 and LF8;
- for the right caudal region defined as RF5, RF6, RF7 and RF8.
- Symmetry values:
- left–right symmetry for minimum, maximum and mean thermal values of the cranial region;
- left–right symmetry for minimum, maximum and mean thermal values of the caudal region;
- front–back symmetry for minimum, maximum and mean thermal values.

Saddle:

Thermographic scans were captured of the underside of the saddle. Using a grid reference applied to each thermogram, minimum, maximum and mean temperatures were obtained from each location and processed using Flir software.

Pooled minimum, maximum and mean temperatures:

- for the cranial region defined as L3 and R3;
- for the mid region defined as L2 and R2;
- for the caudal region defined as L1 and R1.
- Symmetry values:
- left—right symmetry for minimum, maximum and mean thermal values of the cranial region;
- left—right symmetry for minimum, maximum and mean thermal values of the mid region;
- left—right symmetry for minimum, maximum and mean thermal values of the caudal region;
- front—back symmetry for minimum, maximum and mean thermal values.

For the symmetry parameters, values closer to zero represent symmetry between the left and right sides of the body. Positive values indicate increased temperature for the left side, negative values indicate a higher temperature for the right side.

### 2.7. Saddle Pressure Data

The saddle peak and mean (kPa) pressure data were collected from 11 consecutive strides per trial with three repeats totaling 33 ± 3 (mean ± SD) in rising trot and 15 consecutive canter strides per trial with three repeats totaling 45 ± 2 in seated canter. The peak and mean pressure (kPa) values for all loaded cells (>2 kPa) were calculated for each stride and stride values were averaged per trial, resulting in three trial-average values per horse and gait. The saddle mat was then split into quadrants, allowing for the quantification of peak and mean pressure differences between the cranial and caudal regions, with positive values indicating increased pressure in the cranial region and negative values indicating increased pressure in the caudal region.

The following peak and mean pressure derived parameters were used for statistical analysis:

- pressures beneath the cranial aspect of the saddle defined as rows 1–8 and columns A–H (left side) and I–J (right side).
- pressures beneath the caudal aspect of the saddle defined as rows 9–16 and columns A–H (left side) and I–J (right side).
- left-to-right saddle pressure symmetry for the cranial region defined as rows 1–8 and columns A–H (left side) and I–J (right side).
- left-to-right saddle pressure symmetry for the caudal region (left—right) defined as rows 9–16 and columns A–H (left side) and I–J (right side).
- front-to-back pressure differences between cranial and caudal regions (front-back) defined as cranial (rows 1–8, columns A–J)– caudal (rows 9–16, columns A–J).

For the symmetry parameters, values closer to zero represent symmetry between the left and right sides of the body. Positive values indicate an increased temperature for the left side, negative values indicate a higher temperature for the right side.

### 2.8. Statistical Analysis

Statistical analysis was performed in SPSS (vers. 22, IBM, Armonk, NY, USA). Six general linear mixed models were implemented for the minimum, maximum and mean thermographic values as outcome variables. Exercise (baseline, post lunge, post ridden) and saddle fit (correct, narrow, wide) were defined as fixed factors and horse was defined as a random factor, with a Bonferroni post hoc analysis being carried out to determine differences between conditions (exercise: baseline, post lunge, post ridden and saddle fit: correct, narrow, wide). The estimated marginal mean (EMM) and standard error (SE) values are presented from the six general mixed model. Saddle pressure data (correct, narrow, wide) and thermal data from the underside of the saddle (front, mid, back) were analysed using an ANOVA with Tukey post hoc analysis and presented as mean and standard deviation (mean ± SD). To check the normality of the data for the general linear mixed models, histograms of residuals were inspected visually and for the ANOVA data, a Shapiro–Wilk normality test was used to determine data distribution. For all models (saddle pressure and thermography) the significance level was set at *p* ≤ 0.05. Instead of applying the Bonferroni correction to the significance level, alpha, this study reported the Bonferroni adjusted *p*-values (*p*-values based on Fisher’s least significant difference (LSD) multiplied by the number of comparisons carried out). This allows for the assessment of significance with reference to the traditional alpha of 5%, without increasing type II errors.

## 3. Results

### 3.1. Horse Inclusion

All eight horses were in competitive work (Foxhunter or above, British Show Jumping), ranged in height at the withers from 1.60 to 1.70 m with (mean ± SD) of 1.65 ± 0.03 m, had a body mass between 490 and 603 kg (561 ± 23 kg) and were aged nine to 12 years (10 ± 2 years). Each horse underwent a visual lameness evaluation on the day preceding the experiment, performed by an experienced veterinary surgeon. All horses were deemed to be non-lame and did not show any signs of back discomfort. From the objective movement asymmetry measures, horses had (mean ± SD) asymmetry values (mm): HDmin, 4.0 ± 1.4 and HDmax, 5.5 ± 1.0, PDmin 1.3 ± 1.0 and PDmax 4.0 ± 1.1, and HHD 2.4 ± 2.2.

### 3.2. Saddle Fit

Saddles were all jumping type and wool flocked. From the static and dynamic assessment, following the SMS guidelines, *n* = 3 saddles were found to be too narrow, *n* = 3 too wide and *n* = 2 assessed as a correct fit.

### 3.3. Thermographic Data of the Thoracic Region

#### 3.3.1. Minimum Temperatures (°C)

Time point fixed factor: differences between the minimum temperatures were found for the right cranial region (*p* = 0.05) and left caudal region (*p* = 0.04). For the right cranial region, post hoc analysis showed an increase in minimum temperatures post lunge estimated marginal mean (EMM) and standard error (SE), (21.7 °C, (0.8), (*p* = 0.05)) compared to baseline (18.7 °C (0.8)) temperatures. For the left caudal region, an increase in minimum temperatures (23.4 (0.9)), (*p* = 0.04) compared to baseline (20.6, (0.9)) was found. Post hoc analysis did not identify any differences between post lunge and post ridden exercise (*p* = 0.51). Saddle fit fixed factor: no differences were found between saddle conditions (*p* = 0.29) (Table 1).

#### 3.3.2. Maximum Temperatures (°C)

Time point fixed factor: differences were found between the maximum temperatures of the left cranial region (*p* ≤ 0.0001) and right cranial region (*p* ≤ 0.0001). Differences between the maximum temperatures for the left caudal region (*p* = 0.001) and the right caudal region (*p* = 0.001) were found. In all areas, temperatures were higher post lunge and post ridden when compared to the baseline. No differences were found in either the cranial (*p* = 0.12) or caudal (*p* = 0.38) symmetry parameters.

Saddle fit fixed factor: differences in the maximum temperatures were found for saddle widths (*p* = 0.05) however, post hoc analysis did not identify any differences between saddle widths (Table 1).

#### 3.3.3. Mean Temperatures (°C)

Time point fixed factor: differences were found between the mean temperatures of the left cranial region (*p* = 0.003) and right cranial region (*p* = <0.0001). Differences between the mean temperatures for the left caudal region, (*p* = 0.003) and right caudal region (*p* = 0.006) were found. In all areas, temperatures were higher post lunge and post ridden when compared to the baseline. No differences were found in either the cranial (*p* = 0.14) or caudal (*p* = 0.32) symmetry parameters.

Saddle fit fixed factor: no differences were found between saddle conditions (*p* ≥ 0.29) (Table 1).

Table 1 displays the estimated marginal mean (EMM) and standard error (SE) for the minimum, maximum and mean thermal temperatures (°C) of the cranial left/right and caudal left/right region of the thoracic spine along with symmetry values for the left and right sides. Thermographic data were collected from each time point: baseline (BL); post lunge (PL); and post ridden (PR) of eight horses. Positive values indicate an increased temperature value for the left side and negative values indicate a higher temperature value for the right side of the thoracic region. For the time point fixed factor, differences in maximum temperatures of the left cranial (*p* ≤ 0.0001), right cranial (*p* ≤ 0.0001), left caudal (*p* = 0.001) and right caudal area (*p* = 0.001) of the thoracic region were found. Differences in the mean temperatures of the left cranial (*p* = 0.003), right cranial (*p* ≤ 0.0001), left caudal (*p* = 0.003) and right caudal area (*p* = 0.006) of the thoracic region were found. In all regions, post hoc analysis showed temperatures post lunge and post ridden were increased when compared to the baseline temperatures (all *p* ≤ 0.03). Bold indicates significant values *p* ≤ 0.05. Bold figures represent significant differences *p* ≤ 0.05.

### 3.4. Thermographic Data of the Underside of the Saddle

**Figure 4:**
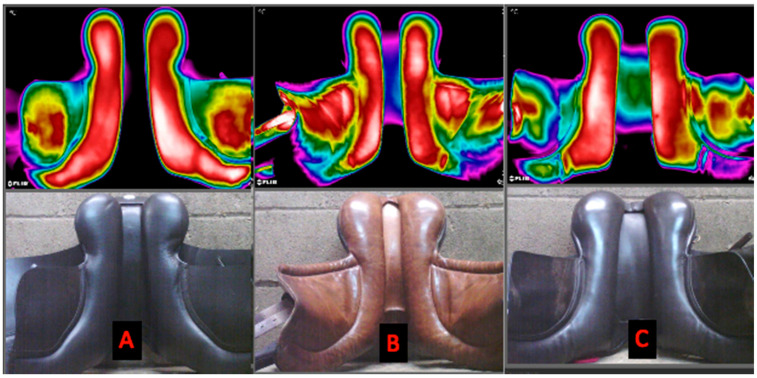
Illustrating thermograms of the ventral aspect of the saddle. (**A**) = correct saddle width, (**B**) = narrow saddle width and (**C**) = wide saddle width. Thermographs taken from three horses who had the highest mean and peak saddle pressures (kPa) during the standardised ridden exercise test. For saddle fit fixed factor: differences were found for the minimum temperatures for the cranial region (Figure 2) (*p* = 0.04). Differences were found in the asymmetry of the maximum temperatures **(°C)** for the cranial region with an increase in asymmetry between the left and right cranial regions (Figure 3), for the correct saddle width (0.8 ± 0.7) compared to the narrow saddle width (0.1 ± 0.1, *p* = 0.03). No differences were found for the remaining parameters (*p* > 0.06).

Saddle fit fixed factor: a general difference between saddle fits was found for the minimum temperature in the underside of the front of the saddle (*p* = 0.04). However, post hoc analysis did not identify specific pairwise differences between saddle widths. Saddle fit fixed factor: a general difference was found in the maximum temperature for the mid symmetry (difference between the left and right front region) (*p* = 0.04). Post hoc analysis showed an increase in asymmetry in the maximum temperature between the left and right front region, for the correct saddle width (0.8 ± 0.7) compared to the narrow saddle width (0.1 ± 0.1, *p* = 0.03). No differences were found for the remaining parameters (*p* > 0.06) (Table 2, Figure 4).

Figure 4—Thermographs of the underside of the saddle.

### 3.5. Saddle Pressure Data

**Figure 5:**
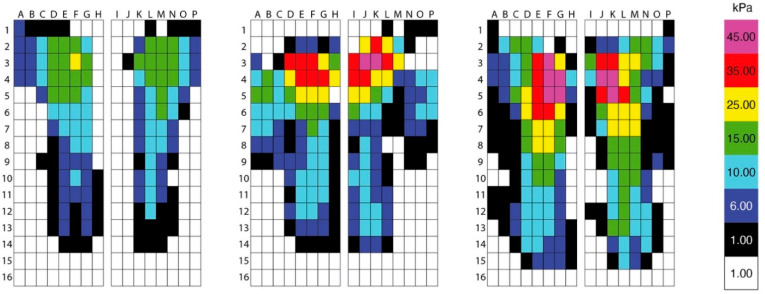
Illustrating pressure distribution beneath three saddles: left = correct saddle width; middle = narrow saddle width; right = wide saddle width from three horses who had the highest mean and peak saddle pressures (kPa) during the standardised ridden exercise test. The mean and peak saddle pressure data were collected during straight-line locomotion from 33 trot strides and 45 canter strides from eight horses ridden by the same rider.

Saddle fit factor: differences in the mean pressures were found between saddle widths for the right caudal region (*p* = 0.03). Post hoc analysis showed an increase in the mean pressures for the wide saddle (9.6 ± 2.2 kPa, *p* ≤ 0.05) when compared to the narrow saddle (5.5 ± 4.2 kPa). Differences in left-to-right differences of the caudal region were found as a function of saddle fit (*p* = <0.0001). Post hoc analysis showed a greater difference between left and right caudal regions for the narrow saddle (−0.4 ± 0.9 kPa, *p* = 0.009) compared to the correct (−2.2 ± 1.0 kPa,) and wide saddle (−2.8 ± 0.9 kPa, *p* ≤ 0.0001). Saddle fit factor: differences were found between the cranial and caudal regions (front–back) (*p* = 0.05) however, no difference was identified after post hoc analysis (Table 3, Figure 5).

Differences in peak pressures were found between saddle widths for the right cranial region (*p* = 0.03). Post hoc analysis showed an increase in peak pressures for the narrow saddle (53.1 ± 13.2 kPa, *p* = <0.04) and wide saddle (53.1 ± 13.3 kPa, *p* = 0.04) compared to the correct saddle width (38.3 ± 2.9 kPa). Saddle fit factor: differences between the cranial and caudal regions (front–back) were found (*p* = 0.01). Post hoc analysis showed an increase in peak pressures for the narrow saddle (31.6 ± 10.8 kPa, *p* = 0.008) and wide saddle (26.7 ± 11.7 kPa, *p* = 0.003) compared to the correct saddle (13.8 ± 4.4 kPa) (Table 3, Figure 5).

Figure 5—Pressure distribution beneath three saddles.

## 4. Discussion

Correct saddle fit is considered essential in order to maintain good back health and allow the thoracolumbar region to function without compromising equine locomotion. Attempting to provide an objective approach, thermography has been proposed as a method of quantifying saddle fit [35,36,37,38,39,40]. Incorrectly fitted saddles, i.e., saddles which are either too wide or too narrow, have been shown to cause focal areas of high pressures in the cranial and caudal thoracic region [2,3]. Given the effect of exercise [41] and the change in the location and magnitude of pressure (as a result of saddle width), the idea that thermal activity may be a useful mechanism for identifying incorrect saddle fit [35,38,39] appears of merit, since the magnitude of pressure, as a result of incorrect saddle fit, may affect blood flow due to capillary occlusion [46]. Thermography has been used to scan the underside of the saddle after exercise [35,36,37] and to quantify differences in thermal patterns between the left and right saddle panels. Thermographic scans have been performed on the horses’ backs immediately after exercise and thermal symmetry of the back was considered a good indicator of saddle fit [39]. Thermal activity has been reported to be increased on the withers and the midline of the back [35,39] and the contact area of the saddle was reported to be asymmetric [35,47] suggesting incorrect saddle fit. Whilst these studies provide partial support for the use of thermography as a means of quantifying saddle fit, they are essentially based on the biological assumption that areas with an increased magnitude of pressure (as a result of saddle fit) will lead to areas of hyperthymic or hypothermic activity as a function of increased or decreased blood flow in the corresponding areas of the horse’s back.

The aim of the present study was to quantify thermographic activity and saddle pressure distribution in a group of non-lame sports horses ridden by the same rider following a standardised thermography protocol including a dynamic exercise test (from here on referred to as: lunge test) and a standardised ridden test. The authors appreciate that this study is limited by its sample size—in particular with respect to saddle fit—and caution should hence be taken when generalising the findings being presented here. However, to the authors’ knowledge, this is the first study to quantify thermal activity and saddle pressure distribution in the same horses. The findings being presented here should be used to advance our understanding of the applications of thermography and its limitations within saddle fitting and provide a basis for future research. In the current study, all thermographic scans were performed by a trained and experienced thermographer. Camera angle and distance from the horses was standardised to limit error; a 20° change in camera angle relative to the object has been shown previously not to affect thermal data [45]. The horse’s thoracic area was not clipped, and the ambient temperature varied (3 °C) and this should be considered when interpreting the results. However, in accordance with scanning requirements, strict protocols (i.e., patient preparation for scanning, enclosed area free from sunlight and draughts, scanning technique—distance and angle) were adhered to, to ensure optimal scanning conditions and thus limit artefacts on the data arising from external factors.

To determine whether any changes in thermal activity were observed as a result of exercise, or the addition of the saddle+rider, a lunge test was performed on a 20 m circle without the saddle or rider prior to the ridden exercise (with saddle+rider). In accordance with our first experimental hypothesis, differences were found between the baseline thermographic and thermal scans obtained directly after lunging. Thermal activity in all regions of the thoracic area (left/right cranial and left/right caudal) (Table 1) were increased as a function of exercise. It seems reasonable to expect such an increase given the physiological response to exercise [41] and increased blood flow to muscles. Across all horses, the minimum, maximum and mean temperatures obtained during the baseline thermogram were highest in the cranial–mid (T10–T14) region of the thoracic spine compared to the caudal region [41]. The reason for this warrants further investigation.

Lunging a horse whilst wearing a saddle has been suggested as a method to evaluate saddle fit [38]. In that study, the underside of the saddle was assessed immediately after lunge exercise [38]. In the current study, we chose not to fit a saddle while the horses were being lunged as our aims were (1) to investigate whether there is an increase in thermal activity as a function of exercise and (2) to determine any differences—induced by adding a saddle+rider—between the lunge test and the standardised ridden exercise test.

In the current study, saddle fit was assessed by five SMS qualified saddle fitters following the SMS’s static and dynamic saddle fitting guidelines. Saddles were found to vary in fit, with the majority of saddles being classified as incorrectly fitting, with three saddles assessed as a wide fit and three saddles assessed as a narrow fit. The inclusion of horses with varying saddle fit provided a “real life” opportunity to compare thermography and saddle pressure measurements to describe saddle fit. In order to limit the influence of inter-horse differences, further studies should be conducted with saddles of different fit used in the same horses in a repeated measures design [2,3,5,6,7,8].

Being aware of the consequences of the small sample size, in particular in relation to a reduced power to detect small differences, an equivalence test [48] for the thermographic symmetry parameters obtained after lunging and after ridden exercise (see Table 1) was performed following the procedure, implementing two one-sided tests of equivalence for paired-samples (TOST-P) [48]. We chose a tolerance value of ±2°, effectively considering changes between post-lunge and post-ridden exercise of less than that amount to be evidence of no change. This tolerance level is consistent with [37]. All one-sided tests indicated that the post-lunge and post-ridden thermographical symmetry outputs can be seen as equivalent (all *p* ≤ 0.027).

Compared to a correctly fitted saddle (determined by the lowest overall saddle force), both the magnitude and location of pressure have been shown to change when horses were ridden on a treadmill in walk and trot [3]. In a narrow saddle, increased pressures were found in the caudal third of the saddle. In the wide and very wide saddles, high pressures were reported for the middle transversal third of the saddle, close to the midline of the equine spine [3]. In an over-ground study, in trot and canter, when ridden in a saddle which was one width fitting wider than correct width fitting (determined by the SMS static and dynamic guidelines and lowest overall force), areas of high pressures were found in the cranial region of the saddle [2]. Measurable concavities have been reported in the epaxial musculature in the region of the 13th thoracic vertebra after 20 min of exercise in a wide saddle, as a function of the magnitude of pressure and its relationship with the epaxial musculature [2]. In the current study, during ridden exercise, the saddles which were classified as a correct saddle fit showed a uniformed pressure distribution. In the saddles classified as too narrow and too wide, the magnitude of pressure was increased in the cranial region of the saddle (difference between the cranial and caudal peak pressures: correct 13.8 ± 4.4 kPa; narrow 31.6 ± 10.8; wide 26.7 ± 11.7). The magnitude of the peak and mean pressure in these cases exceeded the pressures that have been suggested to cause back discomfort [49]. Our findings, in a small sample of horses and saddles, are in accordance with previous studies indicating increased magnitude of pressure in the cranial region when ridden in a wide saddle [2,3].

To limit the effect that different riders may have on saddle pressures (and consequently potentially on thermal activity) we used one experienced show jumping rider [16]. Further studies should ideally make use of a repeated measures design with respect to both saddle and rider, i.e., generating multiple assessments with different saddles and riders in the same horses. In the current study, when using the narrow saddle, the magnitude of pressure was increased in the cranial region, which is different to the findings of previous studies [2,3]. This might be explained by the rider’s position and in particular the rider’s posture indicative of a forward shift of the centre of mass. It may be speculated that this position applies more pressure on the cranial region of the back.

High saddle pressures may lead to hyperthermic or hypothermic activity as a result of altered blood flow, leading to capillary restriction due to increased pressure [46]. In accordance with our second hypothesis associating hyper/hypothermographic areas with areas of increased/decreased magnitude in pressure, areas of high pressures were found in the cranial region for the narrow and wide saddles, but this was not reflected in thermal activity in the cranial (left/right) region or thermal symmetry parameters of the cranial and caudal regions of the thoracic region. We therefore refute our second hypothesis, that areas with increased magnitude of pressure would result in hyper- and/or hypothermic areas. Hyperthermic activity has been suggested to represent high friction or pressure points and hypothermic activity has been suggested to represent intense muscle spasm or severe pressure damage/swelling caused by the saddle [38].

In humans, pressures at surface level penetrate the tissue until they converge on the underlying bony structures, reaching higher values than at the level of the skin surface [50]. The mean capillary pressure in humans is approximately 3.33 kPa [51]. When an external pressure is applied, it increases to >4.26 kPa, at which point blood vessels will restrict and become occluded. In the equine model, saddle pressures are transient and alter with limb movement [6,52,53,54]. It is generally accepted that pressures which are prolonged and exceed mean pressures >11 kPa and peak pressures > 30 kPa [15] are thought to be undesirable and have the potential to cause back discomfort. It seems reasonable to assume that the magnitude of mean (±SD) and peak (±SD) pressures measured in the current study, which for the narrow saddle were 20.2 (±6.3) kPa and 53.1 (±13.2) kPa, respectively, for the wide saddle were 25.1 (±7.4) kPa and 53.1 (±13.3) kPa, respectively, could affect capillary pressure and consequently thermal activity.

No differences were found in thermal activity between the unridden and unsaddled lunge test and the ridden test, and our additional equivalence testing indicated that these conditions are equivalent based on a +/−2° tolerance level. This highlights the importance of performing a standardised unridden dynamic exercise test without a load (saddle) to ensure that exercise-induced changes in thermal patterns are not misinterpreted as being a result of saddle fit [38]. The increase in thermal activity in the thoracic region found in the present study was as a function of exercise and not saddle fit. We hence refute our third hypothesis, which predicted differences in thermographic patterns between the lunge test without a saddle and the ridden exercise test. Our study highlights that, when conducting a dynamic saddle fit test (which necessitates the horse to exercise), the challenges of distinguishing between changes in thermal patterns solely related to exercise and changes related to saddle fit should not be underestimated.

Lastly, thermographs of the underside of the saddle have been proposed as a method which is sensitive enough to determine saddle fit [35,36,37] with differences >2 °C representing incorrect saddle fit [37]. In the current study, the narrow and wide saddles were classified as an incorrect fit based on the SMS Saddle Fitting Guidelines. The thermograms of the underside of the saddle did not appear to reflect the findings of the saddle fit assessment: for both the wide and narrow saddle, mean and maximum thermal asymmetry between the left and right sides were <0.5 °C despite the high pressures observed during ridden exercise. Minimum asymmetry temperature values for the narrow saddle were −2.3 (±4.9) °C, which would appear to be of significance [37]. However, this asymmetry was observed in the caudal region which, from our kinetic saddle data, was not the region of the back experiencing high dynamic pressures. In contrast, in the cranial region, with high pressures observed, the thermal asymmetry was 0.2 (±0.5) °C. Therefore, based on the findings presented here, we refute our fourth hypothesis and recommend that caution is taken when applying thresholds and basing saddle fitting results solely on thermograms of the underside of the saddle.

## 5. Conclusions

This study has investigated the relationship between saddle pressures and thermal activity in the context of saddle fitting. Our findings did not provide evidence supporting a direct link between thermal activity and areas with an increased magnitude of pressure under the saddle. We did, however, find consistent exercise-induced changes in thermal activity. This complicates the use of thermal imaging for assessing dynamic saddle fitting.

## Figures and Tables

**Table 1:** Minimum, maximum and mean thermal temperatures of the cranial and caudal regions of the thoracic spine. EMM: Estimated marginal means; SE: standard error.

	Baseline (BL) (°C) EMM (SE)	Post Lunge (PL) (°C) EMM (SE)	Post Ridden (PR) (°C) EMM (SE)	Exercise Main Effects *p*-Value	Pairwise Bonferroni Post Hoc *p ≤* 0.05	Correct Saddle Width (°C) EMM (SE)	Narrow Saddle Width (°C) EMM (SE)	Wide Saddle Width (°C) EMM (SE)	Saddle Width Main Effects *p* Value	Pairwise Bonferroni Post Hoc *p* ≤ 0.05
Minimum Temperature (°C)										
Left Cranial Region	19.9 (0.7)	22.3 (0.7)	20.8 (0.7)	0.12	-	20.3 (0.9)	20.6 (0.7)	22.2 (0.7)	0.28	-
Right Cranial Region	18.7 (0.8)	21.7 (0.8)	20.3 (0.8)	0.05	BL < PL, *p* = 0.05	20.4 (1.1)	19.1 (0.8)	21.2 (0.8)	0.29	-
Left Caudal region	20.6 (0.9)	23.4 (0.9)	22.4 (0.9)	0.04	BL<PL, *p* = 0.04	21.1 (1.3)	22.5 (1.1))	22.9 (1.1)	0.59	-
Right Caudal Region	20.4 (1.0)	22.9 (1.0)	21.9 (1.0)	0.05	-	21.1 (1.7)	21.7 (1.4)	22.5 (1.4)	0.81	-
Cranial Region Symmetry (left–right)	1.2 (0.5)	0.5 (0.5)	0.5 (0.5)	0.58	-	−0.1 (0.6)	1.5 (0.5)	0.9 (0.5)	0.24	-
Caudal Region Symmetry (left–right)	0.1 (0.2)	0.5 (0.2)	0.5 (0.2)	0.29	-	−0.0 (0.4)	0.7 (0.3)	0.4 (0.3)	0.47	-
Difference between cranial and caudal	−1.1 (0.5)	−1.1 (0.5)	−1.5 (0.5)	0.22	-	−0.6 (1.0)	−2.2 (0.8)	−0.9 (0.8)	0.45	-
Maximum Temperatures (°C)										
Left Cranial Region	26.7 (0.5)	30.9 (0.5)	29.7 (0.5)	<0.0001	BL < PL, *p* = <0.0001 BL < PR, *p* = 0.002	28.5 (0.6)	28.1 (0.5)	30.6 (0.5)	0.05	-
Right Cranial Region	26.9 (0.4)	29.7 (0.4)	29.9 (0.4)	<0.0001	BL < PL, *p* = 0.001 BL < PR, *p* = <0.0001	28.1 (0.8)	27.9 (0.6)	30.0 (0.6)	0.16	-
Left Caudal region	26.9 (0.4)	29.5 (0.4)	30.1 (0.4)	0.001	BL < PL, *p* = 0.005 BL < PR, *p* = 0.002	28.1 (0.6)	28.6 (0.5)	29.6 (0.5)	0.28	-
Right Caudal Region	26.9 (0.4)	29.8 (0.4)	30.1 (0.4)	0.001	BL < PL, *p* = 0.002 BL < PR, *p* = 0.001	28.5 (0.6)	28.7 (0.5)	29.6 (0.5)	0.40	-
Cranial Region Symmetry (left–right)	0.7 (0.3)	0.5 (0.3)	−0.1 (0.3)	0.12	-	0.4 (0.3)	0.2 (0.3)	0.6 (0.3)	0.71	-
Caudal Region Symmetry (left–right)	−0.0 (0.1)	−0.3 (0.1)	−0.1 (0.1)	0.38	-	−0.3 (0.2)	−0.7 (1.6)	0.2 (0.1)	0.34	-
Difference between cranial and caudal	−0.6 (0.3)	0.9 (0.3)	−0.2 (0.3)	0.006	BL < PL, *p* = 0.006 PL > PR, *p* = 0.04	0.3 (0.3)	−0.6 (0.2)	0.6 (0.2)	0.05	-
Mean Temperatures (°C)										
Left Cranial Region	23.1 (0.6)	26.3 (0.6)	25.2 (0.6)	0.003	BL < PL, *p* = 0.003 BL < PR, *p* = 0.03	24.4 (0.9)	24.5 (0.8)	25.7 (0.8)	0.53	-
Right Cranial Region	22.1 (0.6)	26.1 (0.6)	24.9 (0.6)	<0.0001	BL < PL, *p* ≤ 0.0001 BL < PR, *p* = 0.006	24.6 (0.9)	23.3 (0.8)	25.3 (0.8)	0.29	-
Left Caudal region	23.3 (0.6)	26.1 (0.6)	26.2 (0.6)	0.003	BL < PL, *p* = 0.008 BL < PR, *p* = 0.006	24.2 (1.1)	25.1 (0.8)	26.2 (0.8)	0.40	-
Right Caudal Region	23.3 (0.8)	25.8 (0.8)	26.0 (0.8)	0.006	BL < PL, *p* = 0.01 BL < PR, *p* = 0.01	24.5 (1.4)	24.7 (1.1)	26.0 (1.1)	0.67	-
Cranial Region Symmetry (left–right)	0.9 (0.3)	0.1 (0.1)	0.3 (0.30	0.14	-	−0.1 (0.6)	1.2 (0.5)	0.4 (0.5)	0.29	-
Caudal Region Symmetry (left–right)	−0.0 (0.2)	0.2 (0.2)	0.2 (0.2)	0.32	-	−0.2 (0.4)	0.4 (0.3)	0.2 (0.3)	0.45	-
Difference between cranial and caudal	0.7 (0.3)	−0.2 (0.3)	1.0 (0.3)	0.006	BL > PL, *p* = 0.02 PL < PR, *p* = 0.008	−0.1 (0.4)	0.9 (0.3)	0.6 (0.3)	0.22	-

**Table 2:** Minimum, maximum and mean thermal temperatures of the underside of the saddle.

	Correct Saddle Width (°C) Mean ± SD	Narrow Saddle Width (°C) Mean ± SD	Wide Saddle Width (°C) Mean ± SD	Saddle Width Main Effects (ANOVA) *p* Value	Pairwise Tukey Post Hoc *p* ≤ 0.05
Minimum Temperature (°C)					
Cranial Region (left and right)	19.6 ± 2.5	17.8 ± 1.6	19.2 ± 0.6	**0.04**	-
Mid Region (left and right)	19.4 ± 0.9	17.2 ± 1.5	20.1 ± 0.8	0.06	-
Caudal Region (left and right)	20.8 ± 1.4	17.2 ± 2.5	20.8 ± 1.5	0.13	-
Cranial Symmetry (Difference between left and right, cranial region)	0.6 ± 1.4	0.2 ± 0.5	−0.7 ± 0.2	0.19	-
Mid Symmetry (Difference between left and right, mid region)	2.7 ± 4.1	0.6 ± 1.5	1.2 ± 1.1	0.62	-
Caudal Symmetry (Difference between left and right, caudal region)	2.2 ± 0.7	−2.3 ± 4.9	0.2 ± 0.4	0.33	-
Cranial-caudal symmetry (Differences between front and back)	−1.2 ± 0.3	−1.0 ± 1.9	−1.1 ± 1.6	0.98	-
Maximum Temperature (°C)					
Cranial Region (left and right)	23.6 ± 1.6	22.2 ± 0.5	23.1 ± 1.2	0.32	-
Mid Region (left and right)	23.6 ± 1.6	22.2 ± 0.5	23.1 ± 1.2	0.46	-
Caudal Region (left and right)	24.2 ± 1.6	22.5 ± 0.3	23.4 ± 1.4	0.35	-
Cranial Symmetry (Difference between left and right, cranial region)	24.2 ± 1.5	22.9 ± 0.1	23.8± 1.7	0.53	-
Mid Symmetry (Difference between left and right, mid region)	0.8 ± 0.7	−0.4 ± 0.2	0.1 ± 0.1	**0.04**	Correct > narrow, *p* = 0.03
Caudal Symmetry (Difference between left and right, caudal region)	−0.6 ± 0.8	−0.1 ± 0.9	−0.3 ± 0.3	0.81	-
Cranial-caudal symmetry (Differences between front and back)	−0.4 ± 0.7	0.2 ± 0.7	−0.2 ± 0.3	0.53	-
Mid Region (left and right)	−0.3 ± 0.1	−0.5 ± 0.3	−0.5 ± 0.4	0.89	-
Mean Temperature (°C)					
Cranial Region (left and right)	22.6 ± 2.1	20.1 ± 0.8	21.8 ± 1.1	0.67	-
Mid Region (left and right)	22.6 ± 2.1	20.1 ± 0.8	21.8 ± 1.1	0.16	-
Caudal Region (left and right)	22.8 ± 1.9	20.5 ± 0.6	22.4 ± 1.3	0.16	-
Cranial Symmetry (Difference between left and right, cranial region)	23.7± 1.5	21.7 ± 0.2	22.8 ± 1.5	0.30	-
Mid Symmetry (Difference between left and right, mid region)	0.7 ± 0.3	−0.3 ± 0.4	0.1 ± 0.3	0.08	-
Caudal Symmetry (Difference between left and right, caudal region)	0.6 ± 0.7	−0.2 ± 0.4	0.3 ± 0.3	0.26	-
Cranial-caudal symmetry (Differences between front and back)	−0.2 ± 0.5	−0.1 ± 0.3	0.5 ± 0.4	0.21	-
Mid Region (left and right)	−0.1 ± 0.4	−1.4 ± 0.7	−0.6 ± 0.7	0.43	-

Table 2—displaying mean ± S.D. for the minimum, maximum and mean temperatures (°C) of the ventral aspect of the saddle taken immediately after a 20-min standardised exercise test. Differences were found in the maximum temperatures of the mid ventral saddle region (*p* = 0.04). Bold figures represent significant differences *p* ≤ 0.05. Main effects *p*-value was obtained by ANOVA.

**Table 3:** Mean and peak saddle pressures (kPa) during a standardised exercise test.

	Correct Saddle Width (kPa) Mean ± SD	Narrow Saddle Width (kPa) Mean ± SD	Wide Saddle Width (kPa) Mean ± SD	Saddle Width Main Effects (ANOVA) *p* Value	Pairwise Tukey Post Hoc *p* ≤ 0.05
Mean Saddle Pressures (kPa)					
Left Cranial Region	17.2 ± 2.4	20.2 ± 6.3	25.1 ± 7.4	0.06	-
Right Cranial Region	17.5 ± 2.9	20.4 ± 8.5	23.9 ± 6.1	0.19	-
Left Caudal Region	5.1 ± 1.1	5.1 ± 3.5	6.8 ± 2.2	0.31	-
Right Caudal Region	7.4 ± 1.6	5.5 ± 4.2	9.6 ± 2.2	**0.03**	Narrow < wide, *p* = 0.05
Cranial Region Symmetry (Left–Right)	−0.3 ± 1.5	−0.1 ± 2.5	1.1 ± 1.8	0.29	-
Caudal Region Symmetry (Left–Right)	−2.2 ± 1.0	−0.4 ± 0.9	−2.8 ± 0.9	**<0.0001**	Correct > Narrow, *p* = 0.009 Narrow < wide, *p* ≤ 0.0001
Difference between Cranial and Caudal regions (front–back)	11.1 ± 1.3	15.0 ± 5.6	16.2 ± 5.1	**0.05**	-
Peak Saddle Pressures (kPa)					
Left Cranial Region	36.6 ± 2.8	51.5 ± 7.8	51.1 ± 18.1	**0.05**	-
Right Cranial Region	38.3 ± 2.9	53.1 ± 13.2	53.1 ± 13.3	**0.03**	Correct < narrow, *p* = 0.04 Correct < wide *p* = 0.04
Left Caudal Region	21.7±5.1	20.4 ± 6.9	23.4 ± 6.1	0.62	-
Right Caudal Region	25.5 ± 3.1	21.1 ± 8.8	27 ± 7.2	0.20	-
Cranial Region Symmetry (Left—Right)	−1.7 ± 1.6	−1.6 ± 9.2	−2.1 ± 6.7	0.99	-
Caudal Region Symmetry (Left—Right)	−3.8 ± 5.1	−0.5 ± 2.9	−3.8 ± 1.9	0.09	-
Difference between Cranial and Caudal regions (front–back)	13.8 ± 4.4	31.6 ± 10.8	26.7 ± 11.7	**0.01**	Correct < narrow, *p* = 0.008 Correct < wide *p* = 0.003

Displaying mean ± S.D. for the mean and peak saddle pressures (kPa) of the cranial and caudal regions of the saddle. The mean and peak saddle pressure data were collected during straight-line locomotion from 33 trot strides and 45 canter strides from eight horses ridden by the same rider. The mean and peak saddle pressures were obtained for each stride and averaged across each trial. Tukey post hoc analysis was performed with a significance level set at *p* ≤ 0.05. A positive value indicates an increased pressure value for the left side/cranial region, and a negative value indicates increased pressure value in the right side/caudal region. An increase in mean pressures (kPa) for the wide saddle (*p* ≤ 0.05) when compared to the narrow saddle was found. Differences between the cranial and caudal regions (front–back) showed an increase in peak pressures for the narrow saddle (*p* = 0.008) and wide saddle (*p* = 0.003) compared to the correct saddle (Table 3). Bold indicates significant values *p* ≤ 0.05. Main effects p-value was obtained by ANOVA.

## References

[1] Mackechnie-Guire R., Mackechnie-Guire E., Fisher M., Mathie H., Bush R., Pfau T., Weller R. (2018). Relationship Between Saddle and Rider Kinematics, Horse Locomotion, and Thoracolumbar Pressures in Sound Horses. J. Equine Vet. Sci..

[2] Mackechnie-Guire R., Mackechnie-Guire E., Fairfax V., Fisher D., Fisher M., Pfau T. (2019). The Effect of Tree Width on Thoracolumbar and Limb Kinematics, Saddle Pressure Distribution, and Thoracolumbar Dimensions in Sports Horses in Trot and Canter. Animals.

[3] Meschan E.M., Peham C., Schobesberger H., Licka T.F. (2007). The influence of the width of the saddle tree on the forces and the pres-sure distribution under the saddle. Vet. J..

[4] Clayton H.M., Hampson A., Fraser P., White A., Egenvall A. (2018). Comparison of rider stability in a flapless saddle versus a conven-tional saddle. PLoS ONE.

[5] Martin P., Cheze L., Pourcelot P., Desquilbet L., Duray L., Chateau H. (2017). Effects of Large Saddle Panels on the Biomechanics of the Equine Back During Rising Trot: Preliminary Results. J. Equine Vet. Sci..

[6] Murray R., Guire R., Fisher M., Fairfax V. (2017). Reducing Peak Pressures Under the Saddle Panel at the Level of the 10th to 13th Thoracic Vertebrae May Be Associated With Improved Gait Features, Even When Saddles Are Fitted to Published Guidelines. J. Equine Vet. Sci..

[7] Murray R., Mackechnie-Guire R., Fisher M., Fairfax V. (2018). Reducing peak pressures under the saddle at thoracic vertebrae 10–13 is associated with alteration in jump kinematics. Comp. Exerc. Physiol..

[8] Murray R., Mackechnie-Guire R., Fisher M., Fairfax V. (2019). Could Pressure Distribution Under Race-Exercise Saddles Affect Limb Kinematics and Lumbosacral Flexion in the Galloping Racehorse?. J. Equine Vet. Sci..

[9] Belock B., Kaiser L.J., Lavagnino M., Clayton H.M. (2012). Comparison of pressure distribution under a conventional saddle and a tree-less saddle at sitting trot. Vet. J..

[10] Clayton H.M., O’Connor K.A., Kaiser L.J. (2014). Force and pressure distribution beneath a conventional dressage saddle and a treeless dressage saddle with panels. Vet. J..

[11] Latif S.N., Von Peinen K., Wiestner T., Bitschnau C., Renk B., Weishaupt M.A. (2010). Saddle pressure patterns of three different training saddles (normal tree, flexible tree, treeless) in Thoroughbred racehorses at trot and gallop. Equine Vet. J..

[12] Harman J. (1999). Tack and Saddle Fit. Vet. Clin. Am Equine Pract..

[13] Greve L., Dyson S., Pfau T. (2017). Alterations in thoracolumbosacral movement when pain causing lameness has been improved by diagnostic analgesia. Vet. J..

[14] Greve L., Dyson S.J. (2013). An investigation of the relationship between hindlimb lameness and saddle slip. Equine Vet. J..

[15] Byström A., Stalfelt A., Egenvall A., Von Peinen K., Morgan K., Roepstorff L. (2010). Influence of girth strap placement and panel flocking material on the saddle pressure pattern during riding of horses. Equine Vet. J..

[16] Peham C., Licka T., Schobesberger H., Meschan E. (2004). Influence of the rider on the variability of the equine gait. Hum. Mov. Sci..

[17] Dyson S., Carson S., Fisher M. (2015). Saddle fitting, recognising an ill-fitting saddle and the consequences of an ill-fitting saddle to horse and rider. Equine Vet. Educ..

[18] de Cocq P., Clayton H.M., Terada K., Muller M., van Leeuwen J.L. (2009). Usability of normal force distribution measurements to evalu-ate asymmetrical lo.ading of the back of the horse and different rider positions on a standing horse. Vet. J..

[19] de Cocq P., van Weeren P.R., Back W. (2006). Saddle pressure measuring: Validity, reliability and power to discriminate between dif-ferent saddle-fits. Vet. J..

[20] Fruehwirth B., Peham C., Scheidl M., Schobesberger H. (2010). Evaluation of pressure distribution under an English saddle at walk, trot and canter. Equine Vet. J..

[21] Kotschwar A., Baltacis A., Peham C. (2010). The influence of different saddle pads on force and pressure changes beneath saddles with excessively wide trees. Vet. J..

[22] Kotschwar A.B., Baltacis A., Peham C. (2010). The effects of different saddle pads on forces and pressure distribution beneath a fitting saddle. Equine Vet. J..

[23] Alvarez C.B.G., Wennerstrand J., Bobbert M.F., Lamers L., Johnston C., Back W., Van Weeren P.R. (2007). The effect of induced forelimb lameness on thoracolumbar kinematics during treadmill locomotion. Equine Vet. J..

[24] Buchner H.H.F., Schamhardt H., Barneveld A. (1996). Head and trunk movement adaptations in horses with experimentally induced fore- or hindlimb lameness. Equine Vet. J..

[25] Gomez Alvarez C.B., Bobbert M.F., Lamers L., Johnston C., Back W., van Weeren P.R. (2008). The effect of induced hindlimb lameness on thoracolumbar kinematics during treadmill locomotion. Equine Vet. J..

[26] Greve L., Dyson S.J. (2014). The interrelationship of lameness, saddle slip and back shape in the general sports horse population. Equine Vet. J..

[27] Landman M.A.A.M., de Blaauw J.A., Hofland L.J., van Weeren P.R. (2004). Field study of the prevalence of lameness in horses with back problems. Vet. Rec..

[28] Keegan K.G., Dent E.V., Wilson D.A., Janicek J., Kramer J., Lacarrubba A., Walsh D.M., Cassells M.W., Esther T.M., Schiltz P. (2010). Repeatability of subjective evaluation of lameness in horses. Equine Vet. J..

[29] Keegan K.G., Wilson D.A., Wilson D.J., Smith B., Gaughan E.M., Pleasant R.S., Lillich J.D., Kramer J., Howard R.D., Bacon-Miller C. (1998). Evaluation of mild lameness in horses trotting on a treadmill by clinicians and interns or residents and correlation of their assessments with kinematic gait analysis. Am. J. Vet. Res..

[30] Hewetson M., Christley R.M., Hunt I.D., Voute L.C. (2006). Investigations of the reliability of observational gait analysis for the assessment of lameness in horses. Vet. Rec..

[31] Parkes R.S.V., Weller R., Groth A.M., May S., Pfau T. (2009). Evidence of the development of ‘domain-restricted’ expertise in the recogni-tion of asymmetric motion characteristics of hindlimb lameness in the horse. Equine Vet. J..

[32] McCracken M.J., Kramer J., Keegan K.G., Lopes M., Wilson D.A., Reed S.K., Lacarrubba A., Rasch M. (2012). Comparison of an inertial sensor system of lameness quantification with subjective lameness evaluation. Equine Vet. J..

[33] Soroko M., Howell K. (2018). Infrared Thermography: Current Applications in Equine Medicine. J. Equine Vet. Sci..

[34] Schweinitz D.V. (1999). Thermographic diagnosis in equine back pain. Vet. North Clin. N. Am. Equine Pract..

[35] Arruda T.Z., Brass K.E., De La Corte F.D. (2011). Thermographic Assessment of Saddles Used on Jumping Horses. J. Equine Vet. Sci..

[36] Soroko M., Cwynar P., Howell K., Yarnell K., Dudek K., Zaborski D. (2018). Assessment of Saddle Fit in Racehorses Using Infrared Thermography. J. Equine Vet. Sci..

[37] Soroko M., Zaborski D., Dudek K., Yarnell K., Górniak W., Vardasca R. (2019). Evaluation of thermal pattern distributions in racehorse saddles using infrared thermography. PLoS ONE.

[38] Turner T.A., Waldsmith J.K., Wilson J.H. (2004). How to assess saddle fit in horses. Proc. Am. Assoc. Equine Pract..

[39] Masko M., Krajewska A., Zdrojkowski L., Domino M., Gajewski Z. (2019). An application of temperature mapping of horse’s back for leisure horse-rider-matching. Anim. Sci. J..

[40] Soroko M., Jodkowska E., Zabłocka M. (2012). The Use of Thermography to Evaluate Back Musculoskeletal Responses of Young Racehorses to Training. Thermol. Intern..

[41] Witkowska-Pilaszewicz O., Masko M., Domino M., Winnicka A. (2020). Infrared Thermography Correlates with Lactate Concentra-tion in Blood during Race Training in Horses. Animals.

[42] Pfau T., Witte T.H., Wilson A.M. (2005). A method for deriving displacement data during cyclical movement using an inertial sensor. J. Exp. Biol..

[43] Guire R., Weller R., Fisher M., Beavis J. (2017). Investigation Looking at the Repeatability of 20 Society of Master Saddlers Qualified Saddle Fitters’ Observations During Static Saddle Fit. J. Equine Vet. Sci..

[44] Guilds and City Certificate in Saddle Fitting, in Association with the Society of Master Saddlers.

[45] Westermann S., Buchner H., Schramel P., Tichy T., Stanek C. (2013). Effects of infrared camera angle and distance on meas-urement and reproducibilityof thermographically determined temperatures of the distolateral aspects of the forelimbs in horses. J. Am. Vet. Med. Assoc..

[46] von Peinen K., Wiestner T., von Rechenberg B., Weishaupt M.A. (2010). Relationship between saddle pressure measurements and clin-ical signs of saddle soreness at the withers. Equine Vet. J Suppl..

[47] Dantas F., Duarte M., Marins J., Fonseca B. (2019). Thermographic assessment of saddles used in Mangalarga Marchador horses. Arq. Bras. de Med. Vet. e Zootec..

[48] Mara C.A., Cribbie R.A. (2012). Paired-Samples Tests of Equivalence. Commun. Stat.-Simul. Comput..

[49] Nyikos S., Von Rechenberg B., Werner D., Müller J.A., Buess C., Keel R., Kalpen A., Vontobel H.-D., Von Plocki K.A., Auer J.A. (2005). Measurements of saddle pressure in conjunction with back problems in horses. Pferdeheilkunde Equine Med..

[50] Le K.M., Madsen B.L., Barth P.W., Ksander G.A., Angell J.B., Vistnes L.M. (1984). An in-depth look at pressure sores using monolithic silicon pressure sensors. Plast. Reconstr. Surg..

[51] Chang W.L., Seigreg A.A. (1999). Prediction of ulcer formation on the skin. Med. Hypotheses.

[52] Mackechnie-Guire R., Fisher M., Pfau T. (2021). Effect of a Half Pad on Pressure Distribution in Sitting Trot and Canter beneath a Saddle Fitted to Industry Guidelines. J. Equine Vet. Sci..

[53] Mackechnie-Guire R., Mackechnie-Guire E., Bush R., Fisher D., Fisher M., Weller R. (2018). Local Back Pressure Caused by a Training Roller During Lunging With and Without a Pessoa Training Aid. J. Equine Vet. Sci..

[54] Murray R., Guire R., Fisher M., Fairfax V. (2015). A Bridle Designed to Avoid Peak Pressure Locations under the Headpiece and Noseband Is Associated with More Uniform Pressure and Increased Carpal and Tarsal Flexion, Compared with the Horse’s Usual Bridle. J. Equine Vet. Sci..

